# Analysis of Interrelationships among Voluntary and Prosthetic Leg Joint Parameters Using Cyclograms

**DOI:** 10.3389/fnins.2017.00230

**Published:** 2017-04-25

**Authors:** Farahiyah Jasni, Nur Azah Hamzaid, Nor Elleeiana Mohd Syah, Tze Y. Chung, Noor Azuan Abu Osman

**Affiliations:** ^1^Department of Biomedical Engineering, Faculty of Engineering, University of MalayaKuala Lumpur, Malaysia; ^2^Department of Mechatronics Engineering, Kulliyyah of Engineering, International Islamic University MalaysiaSelangor, Malaysia; ^3^Department of Rehabilitation Medicine, Faculty of Medicine, University of MalayaKuala Lumpur, Malaysia

**Keywords:** transfemoral, biomechanics, cyclogram, prosthesis

## Abstract

The walking mechanism of a prosthetic leg user is a tightly coordinated movement of several joints and limb segments. The interaction among the voluntary and mechanical joints and segments requires particular biomechanical insight. This study aims to analyze the inter-relationship between amputees' voluntary and mechanical coupled leg joints variables using cyclograms. From this analysis, the critical gait parameters in each gait phase were determined and analyzed if they contribute to a better powered prosthetic knee control design. To develop the cyclogram model, 20 healthy able-bodied subjects and 25 prosthesis and orthosis users (10 transtibial amputees, 5 transfemoral amputees, and 10 different pathological profiles of orthosis users) walked at their comfortable speed in a 3D motion analysis lab setting. The gait parameters (i.e., angle, moment and power for the ankle, knee and hip joints) were coupled to form 36 cyclograms relationship. The model was validated by quantifying the gait disparities of all the pathological walking by analyzing each cyclograms pairs using feed-forward neural network with backpropagation. Subsequently, the cyclogram pairs that contributed to the highest gait disparity of each gait phase were manipulated by replacing it with normal values and re-analyzed. The manipulated cyclograms relationship that showed highest improvement in terms of gait disparity calculation suggested that they are the most dominant parameters in powered-knee control. In case of transfemoral amputee walking, it was identified using this approach that at each gait sub-phase, the knee variables most responsible for closest to normal walking were: knee power during loading response and mid-stance, knee moment and knee angle during terminal stance phase, knee angle and knee power during pre-swing, knee angle at initial swing, and knee power at terminal swing. No variable was dominant during mid-swing phase implying natural pendulum effect of the lower limb between the initial and terminal swing phases. The outcome of this cyclogram adoption approach proposed an insight into the method of determining the causal effect of manipulating a particular joint's mechanical properties toward the joint behavior in an amputee's gait by determining the curve closeness, C, of the modified cyclogram curve to the normal conventional curve, to enable quantitative judgment of the effect of changing a particular parameter in the prosthetic leg gait.

## Introduction

Human walking is typically characterized by plotting kinematics and kinetics curves as a function of time or percentage of gait cycle. These kinematics and kinetics curves became the primary guideline in the prosthetic leg design that would mimic the normal gait. These were done by ensuring that the prosthesis produces user gait performance that is close to the normal single-variable curve (Engeberg, 2013; Kutilek et al., 2013).

Although it is more common and convenient to use a single variable curve of kinematics or kinetics parameters as guideline in designing the control system for microprocessor based prosthetic leg, literatures proved that there are advantages of analyzing it as a pair. A number of relevant dynamic effects can be identified when pairs of kinematics and kinetic variables are examined together and correlations among them were concurrently assessed (Crenna and Frigo, 2011). These combinations of kinematics and kinetics variables, omitting the time variables from the two signals, create different cyclic trajectories known as cyclograms (Goswami, 1998). From the cyclic trajectories represented by the coupled variables, the dynamic variation of the gait can be easily visualized through the changes in the cyclograms pattern. In addition, the interaction between two co-existing parameter across the joints could be concurrently assessed. This is the foundation of applying the cyclograms concept to determine the most influential knee parameter in producing closest to normal and most efficient gait.

The use of cyclograms over a function of time has been used as a reference for designing a microprocessor-controlled prosthesis (Kutilek et al., 2013, 2014a,b). This is because, the fact that “locomotion is a tightly coordinated movement of several limb segments” can be more naturally grasped as the coupled variables of two or more joints rather than individual joint kinematics or kinetics. The cyclogram pattern was noted to be an extremely stable mechanism to identify gait behavior due to the use of cyclic traces of joint variables (Kutilek et al., 2014a). This was achieved based on the principal that a coordinated motion of a leg is to be perceived as an interaction between two or more limb segments rather than a phenomenon of isolated joint movements over time (Awai and Curt, 2014).

According to Pitkin, designing a better prosthetic leg does not revolve around the integration to mimic close-to-normal limb segments behavior (Pitkin, 2010). The design should take into account the amputees pathological condition (Pitkin, 2010). Therefore, there is a need to outline the basis for the prosthetic design that requires minimal modification to adjust with, if not all, most of the amputees' conditions.

The techniques that had been used to mathematically represent gait data and to analyze it include fuzzy system methods, multivariate statistical analysis, fractal dynamics, artificial intelligence (including neural networks), and wavelet methods (Arjunan and Kumar, 2010). Neural networks had been the most prevalent emerging non-traditional method applied to the analysis of gait data (Su and Wu, 2000). The combination of neural network and cyclograms in a single study is rarely found in the literature, and none has been introduced for identifying the gait disparity in amputees. To date, neural network has been used to perform automated diagnosis of gait patterns represented by angle-angle cyclogram (Barton and Lees, 1997). In one study (Barton and Lees, 1997), once trained, the network can identify three different conditions—normal gait, a gait with unequal leg length and a gait with unequal leg weights at 83.3% success rate. The work of Kutilek and Farkasova that described the method of predicting the motion of lower extremities using neural network and had suggested that the predicted data may be useful for evaluation of human walking in physiotherapy practice based on angle-angle diagram (Kutilek and Farkasova, 2011). Their method was one of the earliest to apply neural network in a clinical practice for study of disorders or characteristics in motion function of human body, and also to be used in new design of lower limb prosthesis (Kutilek and Farkasova, 2011).

This study proposed a method of using the cyclograms as the cyclic representation of locomotion and two-variable interaction, to determine the most influential parameter in each gait sub-phases for the knee control design in transfemoral prosthesis. Using neural network, we analyzed the interaction between the users' gait coupled parameters holistically (of the joint itself and across two joints) toward the least error production from normal profile. The final parameter at each gait sub-phase was then determined by analyzing the effect of manipulating one parameter onto another parameter of the coupled variables at the most deviated cyclogram for different amputee's profile.

## Methods

This study consists of 3 phases. The first phase involved gait analysis and cyclogram model generation based on the leg joint parameters. Phase 2 verified the generated cyclogram models by determining the gait deviation at each sub phase throughout the gait cycle of participants with different pathological conditions. In the last phase, the modified cyclogram models were used to identify the most dominant parameter at each gait sub-phase in the case of transfemoral amputees.

### Phase 1: gait analysis and generating cyclograms

In this section, the data collection procedure was explained in detail. Then, the generation of cyclogram pairs from the voluntary and mechanical joints' parameters was described.

#### Data collection and gait analysis method

Table 1 describes the details of the participants involved in this work.

**Table 1:** **Description of the subjects**.

**Type of subjects**	**No of subjects**	**Mass (kg)**	**Height (cm)**	**Age (years)**	**Criteria**
Normal healthy subjects (served as control group)	20	65.43 ± 17.96	161.4 ± 10.25	24 ± 2.53	No pathological conditions No history of lower limb surgery No physiological disease Right limb dominant Can walk without assistance/any upper extremity aids
Transtibial amputees	10	77.15 ± 21.45	168.45 ± 9.95	45.70 ± 9.9	Unilateral amputee Has been wearing prosthesis for more than 6 months.
Transfemoral amputees	5	70.60 ± 19.07	167.2 ± 7.58	34 ± 7.56	
Orthosis wearer (represent prosthesis users who do not have a perfect prosthetic device)	10	67.40 ± 17.31	165.2 ± 10.81	35.40 ± 12.9	Anatomical joints are still intact Able to walk without wearing orthosis

The profile data for each of the transtibial, transfemoral and orthosis subjects and their respective pathological condition are presented in Table 2. All the subjects provided their written and informed consent by signing the consent form provided to them, to participate in this study. Approval for the gait analysis procedures was obtained from the University Malaya Medical Centre Ethics Review Board and this study confirmed with the regulatory standards.

**Table 2:** **Profile data for transfemoral, transtibial and orthosis subjects**.

**Subject code**	**Reason of amputation/wearing orthosis**	**Type of Prosthesis knee, locking, and foot/orthosis**	**Affected side**
TF1	Osteosarcoma	Mechanical knee joint, Auto-lock system	R
TF2	Diabetes, infection	Quadrilateral socket, single axis knee joint, SACH foot	R
TF3	Trauma	Mechanical knee-joint, single-axis foot	R
TF4	Doctor carelessness during surgery	Hydraulic knee joint, auto-lock system Flex-foot	R
TF5	Trauma	Mechanical knee-joint, flex-foot	R
TT1	Trauma	Pin lock, Flex-foot	R
TT2	Trauma	Shuttle lock, SACH foot	R
TT3	Trauma	Pin lock, SACH foot	L
TT4	Trauma	Shuttle lock, Flex-foot	R
TT5	Trauma	Pin lock, flex-foot	L
TT6	Trauma	Shuttle lock, flex-foot	L
TT7	Diabetes	Shuttle lock, Flex-foot	R
TT8	Trauma	Pin lock, SACH foot	L
TT9	Trauma	Shuttle lock, Flex-foot	R
TT10	Gangrene on 1st toe, Diabetes	Pin lock, Flex-foot	R
OT1	Congenital flexible pes planus	Custom-made shoe with arch insole	B
OT2	Limb length discrepancy (1.2 cm)	Custom-made insole	L
OT3	Diabetes, 1st metatarsal ray amputation	Diabetic shoe	L
OT4	Inflammation at medial collateral ligament	Knee brace	R
OT5	Diabetes, callus at 5th metatarsal	Diabetic shoe with insole	B
OT6	Flexible Pes Planus	Arch insole	B
OT7	Diabetes, 2nd metatarsal ray amputation	Custom-made insole	R
OT8	Flexible pes Planus	Custom-made insole	B
OT9	Hallux valgus, present of bunion on 1st metatarsal	Hinged AFO	L
OT10	Charcot foot	Rigid AFO	R

*R, Right; L, Left; B, Both; TT, Transtibial; TF, Transfemoral; OT, Orthotic; AFO, Ankle-foot Orthosis*.

The experimental procedures of this study were conducted in a motion analysis lab on a 4 m straight walkway. The lab was equipped with an optical three-dimensional motion capture system, a 6-infrared camera Vicon Nexus 1.5 (Vicon, United Kingdom) mounted strategically around the room, working at 50 Hz and integrated with two force plates (Kistler Instruments, Switzerland) working at 200 Hz, located midway to obtain synchronized kinematic and kinetic data within a capture volume of approximately 4 × 4 × 2 m.

The subjects' bilateral leg length (from greater trochanter to lateral malleolus), ankle width and knee width were measured and recorded to the system software for automated calculation of mass, center of mass and moment of inertia. The knee width of residual limb was considered as similar with the sound limb for transfemoral subjects. The ankle width for both transfemoral and transtibial subject residual limb were also considered to be similar to the sound limb measurement. Ground reaction force was obtained from the force plates for kinetic automated calculation by the Vicon software to obtain joint power and moment. Sixteen reflective markers of 14 mm diameter were attached bilaterally based on Helen Hayes marker placement.

At least 20 trials were recorded for each subject and all the subjects were asked to rest in between the trial to prevent fatigue effect to the data collection. The gait data was analyzed with a conventional model Vicon Plug-In-Gait and filtered using second-order Butterworth filter. The ankle, knee and hip joint kinematics, moments and gait events as well as relevant markers' trajectories, Ground Reaction Force (GRF) and Center of Pressure (COP) data were imported into Matlab for extraction and further analysis. Linear interpolation was applied to the original data points to obtain data points for joint kinematics and kinetics data at every 2% of stride duration. The joint moment was computed by inverse dynamics, using subjects' measurements and anthropometric properties and normalized to body weight (Robertson, 2004).

The ankle dorsi-plantar flexion angles, knee and hip flexion-extension angles were the kinematic data required in this study, while for kinetic analysis, all the three joints' sagittal moment and power were extracted.

#### Cyclogram models generation

A total of 36 relationships were obtained by pairing the variables of the joint itself and across the two joints pair. The relationships can be classified into 6 groups, namely:

- angle-angle across the two joints pair (3 pairs),
- moment-moment across the two joints pair (3 pairs),
- power-power across the two joints pair (3 pairs),
- angle-moment within the joint and across the two joints pair (9 pairs),
- angle-power within the joint and across the two joints pair (9 pairs), and,
- moment-power within the joint and across the two joints pair (9 pairs).

Most of these relationships were not found in the literature except for angle-angle relationship across the two joints pair (Cavanagh and Grieve, 1973; Goswami, 1998) and moment-angle of the ankle joint (Crenna and Frigo, 2011; Wang et al., 2012).

The cyclogram relationships for the normal subjects were obtained by averaging parameters of 200 trials from 20 subjects due to the consistency of the gait sub-phase duration among the normal subjects. On the other hand, this averaging method does not apply to the transtibial, transfemoral, and orthosis subjects because of their irregularities of gait sub-phase duration in each trial. Thus, the relationships were obtained from each of the trial, for 10 trials for these amputee subjects. The amputee subjects' cyclograms in addition to its respective gait sub-phases from the trials would be input into the neural network in Phase 2 to predict the output of subject-based movement profiles and identify the gait deviation at each of the gait sub-phase.

From the normal cyclograms relationship obtained, it was found that the geometric shape of the cyclograms changed as the traveling direction of the gait sub-phases change between both legs. Thus, it was deduced that the interaction between the two variables were different between left and right leg. Primarily, this geometric difference was influenced by the limb dominance effect on the normal walking gait. The dominant limb was the preferred limb (leading limb) that the subjects used for mobilizing or propulsion; while in contrast, the non-dominant limb is the non-preferred limb (trailing limb) that is used to support the actions, i.e., stability control. This support and mobility task performed by each leg was interpreted as gait asymmetry that is normal to occur even to healthy able-bodied subjects. All the normal subjects in this study were right limb dominant, therefore, right side served as the propulsion source during walking. The necessity of defining which limb contributes to propulsion or support task was important in this study in order to select the reference side of cyclograms that was identical to the functional role of the prosthetic and the intact side for amputees. It has been revealed in the study by Carpenter et al. that the transtibial amputees who undergone osteomyoplastic amputation (also known as Ertl) rely on the prosthetic side to support, while the intact side function to propel the body forward (Carpenter et al., 2012). This is further supported by the study that revealed the unilateral amputees experience increased asymmetry in their intact limb during loading and stance time due to the loss of ankle plantar-flexors which contribute to body propulsion and swing initiation (Liu et al., 2006). Therefore, the subsequent phases in this study make use of the cyclograms on the left side (non-dominant) to study the deviation that occur on the prosthetic side of the amputee.

### Phase 2: using cyclograms to quantify gait disparity in terms of gait sub-phases

**Figure 1:**
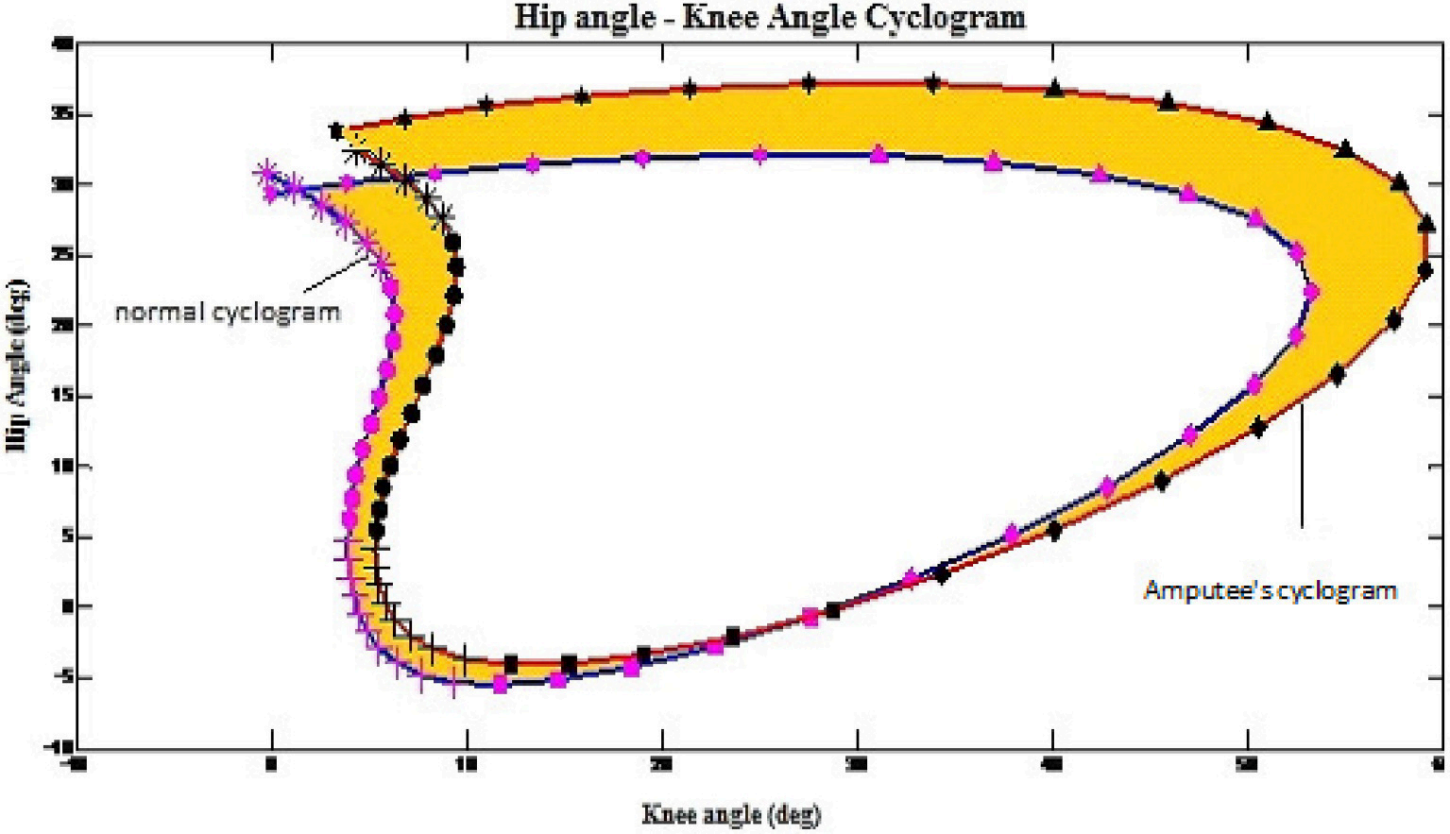
**Example on how the cyclograms being applied to obtain the gait disparity in amputees**. The shaded area indicate disparity from norm.

All 36 cyclograms that were created in previous phase were used to determine the gait disparity of the amputees. Figure 1 illustrates the example on how the cyclograms was utilized to determine the gait disparity occurrence at each gait sub-phase for each of the subject. The orange-colored area represents the error between amputee's cyclogram and the normal cyclogram, i.e., the gait disparity. The higher the error as compared to the normal gait's cyclogram, the higher is the gait deviation of the subject.

At this stage, neural network was adopted as the tool to model the interaction between two parameters in a cyclogram, as well as to calculate the disparity between cyclograms. Hence, the feedforward network with backpropagation algorithm was employed. This network was chosen as it uses supervised learning rule during the training. The supervised learning rule provide an example for the network learning, and the network learn by comparing the predicted output with the desired output, and backpropagate the error to update the network weight/bias (i.e., the constant used to define the strength of the input in determining the output) (Beale et al., 2010). The neural network architecture was constructed in Matlab and the predicted outputs and errors obtained were used to quantify the disparity occurrence at each of the gait sub-phase.

**Figure 2:**
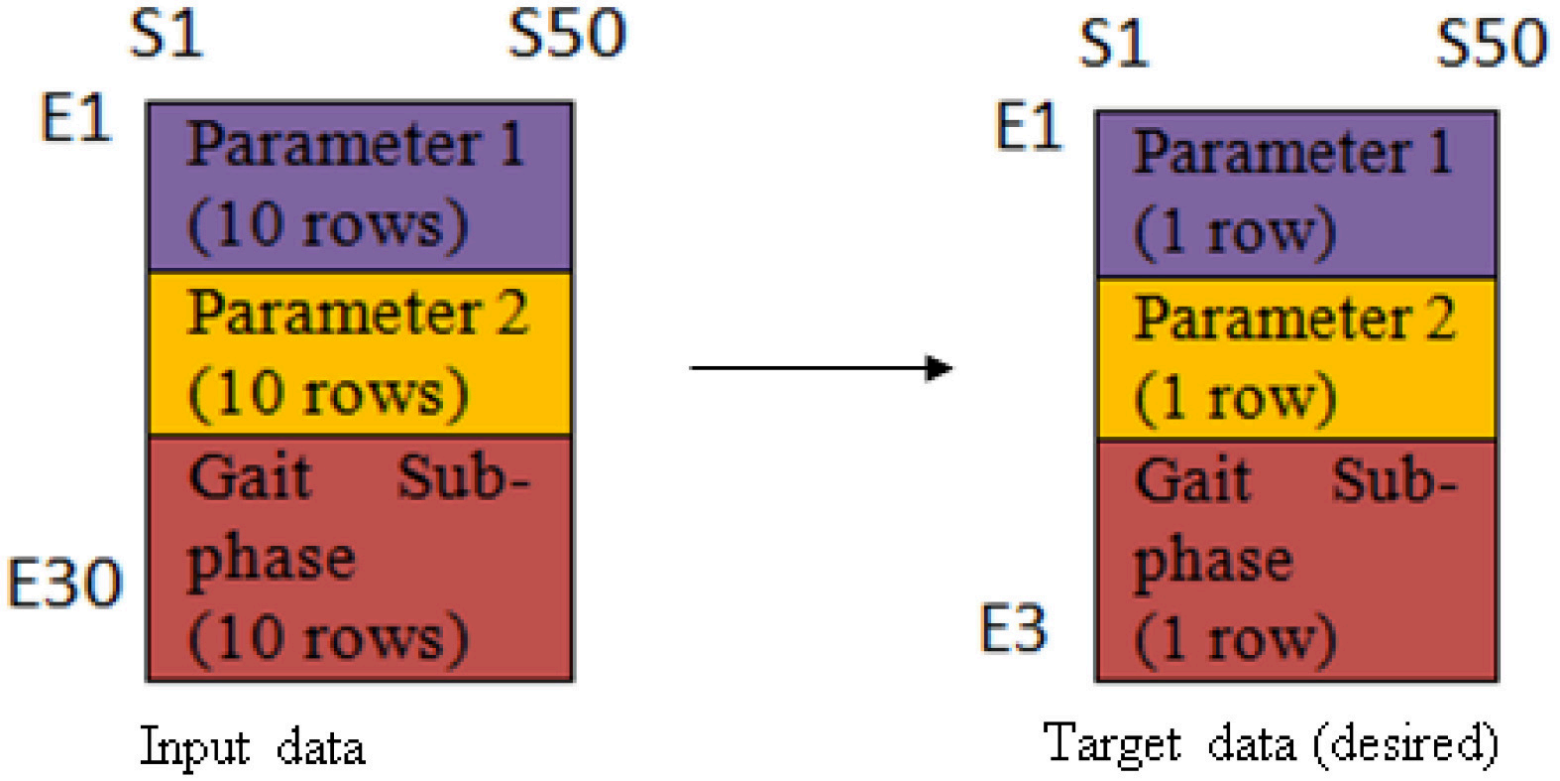
**Illustration of the arrangement of input data and target data for network training**.

The network consisted of 3 layers; input layer (30 neurons), hidden layer (7 neurons) and output layer (3 neurons). The relationship between the input data and the output data was found linear, thus linear regression functions were employed. The input and target data arrangement was illustrated as in Figure 2. The first 10 rows (E1-E10) represent the first cyclogram's variable for 10 trials, the next 10 rows are the second cyclogram's variable for 10 trials, and the last 10 rows are the gait sub-phases which annotated as 1 until 7 corresponding to loading response to terminal-swing for 10 trials. On the other hand, S1-S50 represents the samples of the variables series at an interval of 2% gait cycle and its respective gait sub-phase duration.

The network was first trained with data of 100 trials that was randomly chosen from 10 healthy subjects as network input and average normal data of 200 trials from 20 healthy subjects as the network target in order for the NN to learn the outcome of different paired variables interactions of normal subject as the reference. A total of 36 NN were trained with 36 paired variables, i.e., cyclograms, respectively. During the training, default random division algorithm was used for the NN to divide the data randomly for training (60%), validation (20%), and testing (20%). The network weight was reinitialized if all the following criteria for the network performance were not met, and only network that fulfilled the following criteria were kept for simulation with prosthetic cyclograms: (1) final mean square error <0.9 at the final iteration, (2) the test set error and validation set error has similar characteristics, (3) the regression for all set was > 0.9.

**Figure 3:**
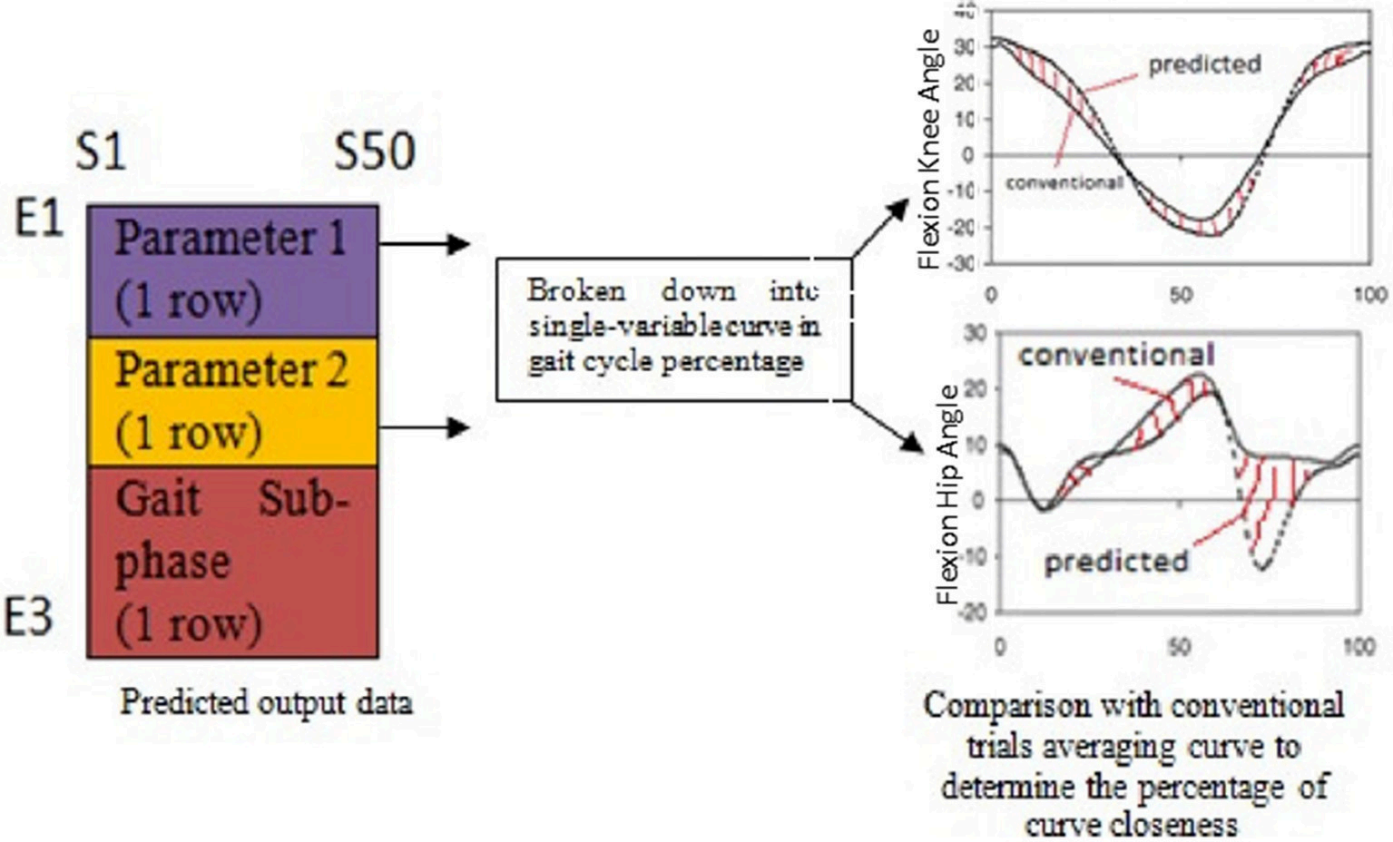
**Workflow to validate the network prediction ability; the errors between the two curves were calculated at each point for each case of the subject**.

Before simulating the trained network with amputee's cyclograms, the network was first tested and validated with 5 random normal subjects and 25 different cases of amputees and orthosis subjects' pathological conditions. The predicted output of cyclogram's pair from the simulation was split into two variables curve as a function of gait cycle percentage. These two curves were then compared with the curves that used the conventional trials averaging method. The errors between the two curves (*e*_*p*−*c*_) were calculated for each subject as illustrated in Figure 3.

The network model prediction ability was presented in terms of percentage of the closeness of the network predicted output curve as compared to the conventional trials averaging curve. The percentage of closeness was calculated as in Equation (1), with *C* = 100% indicates totally close to normal or a perfect match to normal.

(1) $$C=(1-e_{p-c})\times 100$$

where

C = curves closeness

*e*_*p*−*c*_ = Errors between predicted curve points and conventional curve points, with 1 being maximum (100%) error or difference between predicted and conventional curve points, and 0 to be no error at all.

The validation percentage presented in Table 3 indicates that the model, as it was trained with the normal subjects' data, was as expected to have prediction ability of more than 90% with randomly-selected normal subjects. As for orthosis, transtibial, and transfemoral subjects, the percentage range indicates that the NN used has the ability to segregate the deviated data as a whole series of gait cycle and by the two interacting variables that might have contributed most to the gait deviation. The validation for these “pathological” subjects was expected to be <90%, with transfemoral subject having the greatest error (Table 3) due to the least voluntary control of residual limb. This error was used as the quantifying measure in this study.

**Table 3:** **Percentage range of closeness between networks predicted output curve and conventional trials averaging curve**.

**Subjects**	**No. of subjects**	**Percentage range (close-to-conventional-curve), C**
Normal	5	91–99%
Orthosis	10	75–87%
Transtibial	10	73–81%
Transfemoral	5	68–79%

### Phase 3: using manipulated cyclograms to identify the dominant parameter(s) in each gait sub-phases

**Figure 4:**
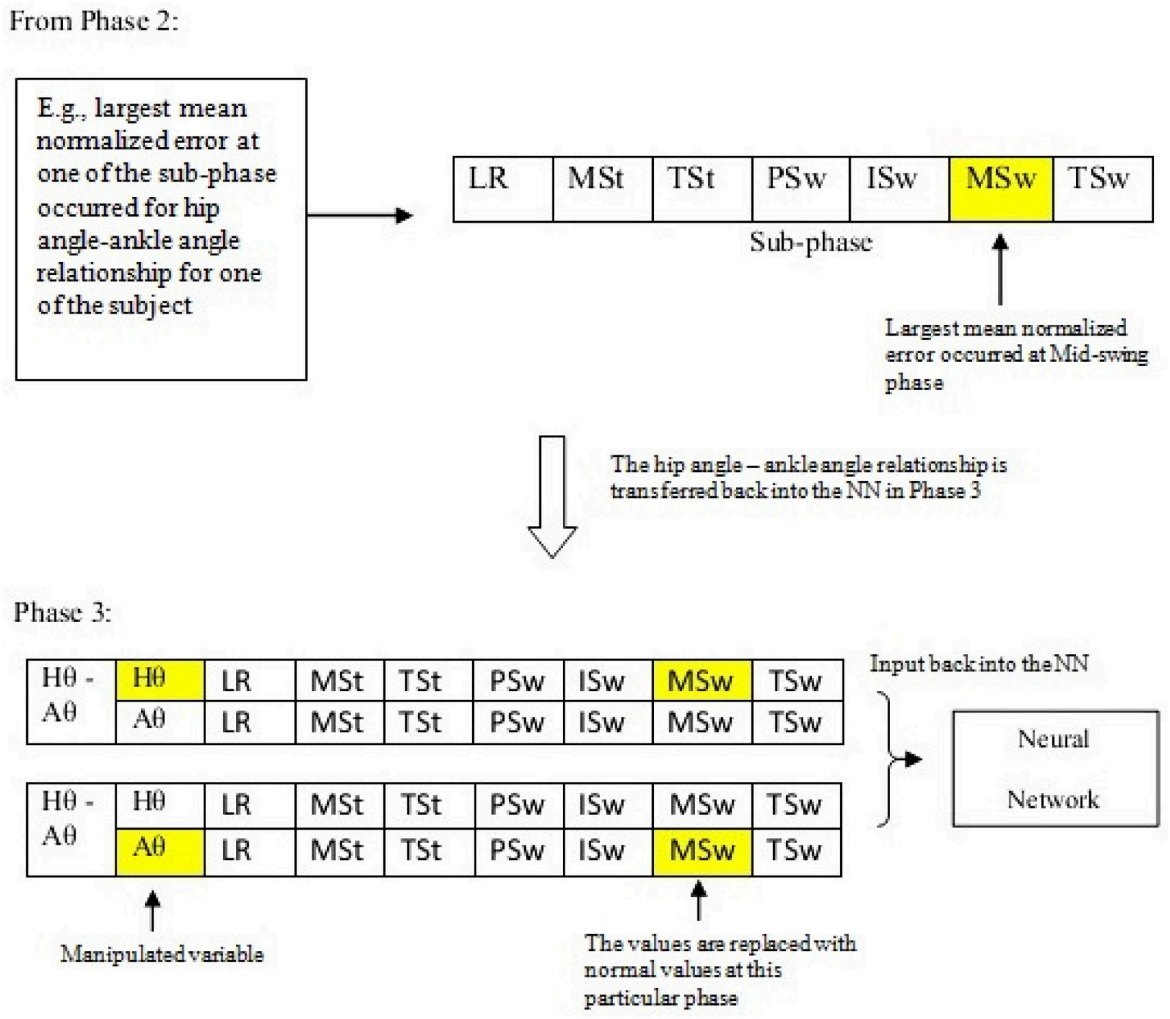
**The workflow of transferring the results from Phase 2 and Phase 3**.

In this phase, the results obtained from the previous phase were used to assess the effect of manipulating one variable toward the other variable of the cyclograms. At each of the gait sub-phase for each amputee, the cyclograms relationship that resulted in the largest deviation was then being input back into the linear network model. A modification of the variable values at the particular sub-phase with the highest mean normalized error was done by inserting the normal value (i.e., from healthy subjects that is averaged using conventional trials averaging method described in Section Cyclogram Models Generation) at one of the paired variables as illustrated in Figure 4. The network model was simulated with the modified input data and the target output data was again being supplied. The error between the predicted output and the desired output was calculated again at each of the sub-phase. This process was repeated with the other variable of the pair. The results obtained were then compared to determine which parameter manipulation gives predicted output that was closer-to-normal cyclograms. This was the crucial identification step in determining the relevant parameter to be controlled at each of the gait sub-phase.

## Results

### Using cyclograms to quantify gait disparity in terms of gait sub-phases

The cyclograms relationship that contributed to the highest mean normalized error at each gait sub-phase for each subject was summarized in Table 4. At loading response (LR), the kinematics-kinetics relationship shows high mean normalized error for each of the subject. The kinetics variable of the pair indicated in red color contributes to the highest mean normalized error, except for subjects TT3 (ankle angle, Aθ) and TT5 (knee angle, Kθ).

**Table 4:** **Summary of the paired variables that contribute to the highest mean normalized error at respective gait sub-phase for each amputee subject**.

**Subjects**	**Cyclograms relationship that contribute to highest mean normalized error**						
	**LR**	**MSt**	**TSt**	**PSw**	**ISw**	**MSw**	**TSw**
TT1	Kθ-AM **(17.72)**	Hθ-HM **(10.88)**	Hθ-HM **(115.16)**	Aθ-KP **(41.40)**	Hθ-HP **(56.37)**	Hθ-HP **(579.25)**	Kθ-AM **(430.12)**
TT2	Hθ-KP **(10.63)**	Hθ-HM **(9.08)**	Hθ-HM **(141.10)**	AM-HP **(140.47)**	AM-HP **(36.37)**	Hθ-HP **(369.53)**	Kθ-KP **(245.60)**
TT3	Aθ-HM **(51.69)**	Aθ-KP **(50.31)**	Hθ-HP **(156.78)**	Aθ-KP **(38.67)**	Aθ-HP **(223.30)**	Aθ-HP **(367.81)**	Kθ-AM **(706.88)**
TT4	Kθ-AP **(13.89)**	Hθ-HM **(6.65)**	Hθ-HM **(104.56)**	Hθ-HP **(64.19)**	Hθ-HM **(50.44)**	Hθ-HP **(455.88)**	Kθ-AP **(191.65)**
TT5	Kθ-KM **(11.16)**	AM-HP **(8.48)**	AM-HP **(114.92)**	Hθ-HM **(49.71)**	Hθ-HP **(1181.79)**	Kθ-AP **(294.89)**	Kθ-AM **(921.14)**
TT6	Hθ-KP **(10.70)**	AM-HP (**14.42)**	AM-HP **(76.40)**	Aθ-HP **(36.98)**	Hθ-HP **(85.94)**	Hθ-HP **(207.19)**	Kθ-AP **(365.22)**
TT7	Hθ-HM **(8.85)**	Hθ-HM **(11.59)**	Hθ-HP **(156.96)**	Kθ-AM **(26.04)**	Hθ-HM **(85.01)**	Hθ-HP **(360.35)**	Kθ-AM **(255.87)**
TT8	Hθ-HM **(13.37)**	AM-HP **(15.46)**	Hθ-HM **(73.98)**	Hθ-HM **(10.24)**	Kθ-AP **(73.85)**	Hθ-HP **(523.18)**	Kθ-AP **(224.12)**
TT9	Aθ-KP **(7.93)**	Hθ-HP **(8.15)**	Hθ-HP **(136.23)**	Hθ-HM **(19.62)**	Hθ-HM **(39.38)**	Hθ-HP **(509.18)**	Kθ-AP **(186.91)**
TT10	Aθ-KP **(9.43)**	Hθ-HM **(9.45)**	Hθ-HP **(125.66)**	Kθ-AM **(22.24)**	Kθ-AP **(69.57)**	Hθ-HP **(404.98)**	Kθ-AP **(349.92)**
TF1	Kθ-AM **(16.11)**	Hθ-HM **(9.50**)	Hθ-HM **(225.66)**	Hθ-HP **(43.74)**	Kθ-AP **(127.43)**	Hθ-HP **(525.05)**	Hθ-Kθ **(402.20)**
TF2	Kθ-AM **(15.17)**	Hθ-HM **(8.33)**	Hθ-HM **(132.96)**	Hθ-HP **(51.44)**	Hθ-HM **(87.00)**	Hθ-HP **(570.20)**	Hθ-Kθ **(499.66)**
TF3	Hθ-HM **(13.46)**	Hθ-HP **(26.83)**	Hθ-HP **(400.53)**	Hθ-HP **(143.48)**	Kθ-AP **(70.98)**	Hθ-HP **(525.00)**	Hθ-Kθ **(489.07)**
TF4	Kθ-AM **(12.71)**	Hθ-HM **(9.71)**	AM-HP **(230.08)**	AM-HP**(87.39)**	Hθ-KM **(33.77)**	Hθ-HP **(520.47)**	Kθ-AP **(502.44)**
TF5	Kθ-AM **(15.52)**	Aθ-KP **(6.71)**	AM-KP **(12.92)**	Hθ-HP **(133.40)**	Hθ-HM **(38.50)**	Hθ-HP **(329.21)**	Kθ-AP **(432.54)**

*A, Ankle; K, Knee; H, Hip; θ, Angle; M, Moment; P, Power*.

*Red-colored acronyms represent the variable correspond to yielding the highest mean normalized error. The bold figures in bracket refer to the mean normalized error of one of the paired variable, Ē*.

At mid-stance (MSt) phase, the result indicated that the kinematics-kinetics relationship has the highest mean normalized error for each of the subject, except for TT5 (AM-HP), TT6 (AM-HP), and TT8 (AM-HP), which shows the kinetics-kinetics relationship. Nevertheless, the variable of the pairs that contributes the highest mean normalized error at this phase were all originated from the kinetics variable (knee power/hip power/hip moment). Except for TT5, TT6, TF4, and TF5 which shows that kinetics-kinetics relationship has high mean normalized error at terminal-stance (TSt), the rest showed that the kinematics-kinetics pair exhibits high mean normalized error. Similar with the MSt phase, the kinetic variable (knee power/hip power/hip moment) of the pairs has the highest mean normalized error in TSt phase compared to the kinematic variable.

Similar cyclograms' relationship profile as the aforementioned MSt and TSt were found for pre-swing (PSw), initial-swing (ISw) and mid-swing (MSw), whereby most of the relationships that yield the high mean normalized error were of kinematics-kinetics pair except for TF4 (AM-HP) in pre-swing and TT2 (AM-HP) in ISw. As for terminal-swing (Tsw), 10 out of the 15 amputee subjects showed unanimously that the kinematics of knee angle variable yield the highest mean normalized error. This contradicted with the results of previous gait sub-phases in which kinetics variable (ankle moment/ankle power/knee moment/knee power/hip moment/hip power) of the pair contributed to the highest mean normalized error.

### Using manipulated cyclograms to identify the most dominant parameter in each gait sub-phase

The results of the manipulated variable that showed the lowest mean normalized error were translated as the variable that induced closest-to-normal cyclograms curve compared to the after-effect of manipulating the other parameters. The overall results were summarized as in Table 5. The results indicated that at the LR phase, when the knee power was manipulated, i.e., replaced with normal values, the mean normalized error was reduced at the phase and consequently the other phases along the gait cycle were also improved. At MSt phase, the result showed that manipulating ankle angle and knee power produced the lowest mean normalized error. The results obtained from NN showed that manipulating either one of these two parameters would yield the same result along the whole gait cycle. This indicated that modifying either one of the parameter is sufficient to produce close to normal gait profile during MSt. For TSt, ankle angle was the dominant parameter that demonstrated the lowest mean normalized error. While at PSw and ISw, hip power and hip angle, respectively produced the lowest mean normalized error.

**Table 5:** **Manipulated parameter that yield the lowest mean normalized error, Ē**.

**Gait Sub-phase**	**LR**	**MSt**	**TSt**	**PSw**	**ISw**	**MSw**	**TSw**
a) Summary of the parameter that yield the lowest mean normalized error	Knee Power	Ankle Angle, Knee Power	Ankle Angle	Hip Power	Hip Angle	-	Knee Power
b) Revised parameter at each of the gait sub-phase specifically for prosthetic knee	Knee Power	Knee Power	Knee Moment, Knee Angle	Knee Angle, Knee Power	Knee Angle	-	Knee Power

*MSw phase does not require any joint control*.

Manipulation of any one of the parameters at MSw does not improve the sub-phase normalized errors. Observation at this phase indicated that the manipulation of parameter at this phase consequently caused the neighboring phases to yield a large mean normalized error. Therefore, no parameter was selected for manipulation at this sub-phase. Finally, for the TSw, knee power parameter was selected as it demonstrated the lowest mean normalized error when being manipulated.

#### Identifying dominant knee parameters for potential microcontroller based knee application: a case study

All the dominant parameters identified through this method were of either hip, knee or ankle parameters. However, in case of minimizing gait disparity through optimization of the knee parameters only, results related to hip and ankle parameters were revisited to identify the dominant knee parameters in its place. This was done by referring to the values of mean normalized error at Phase 2 between hip and ankle variables paired with knee variables. The knee variables that yielded the least mean normalized error when paired with hip and ankle variables of the result as in Table 5(a) was extracted at that particular sub-phase. The revised result, i.e., for knee parameters only, is presented in Table 5(b).

## Discussion and conclusion

This study modeled all cyclograms of lower limb joint's gait variables holistically and analyzed it to identify the gait disparity among prostheses users. It also identified the most dominant knee parameter at each gait sub-phase for a prosthetic knee design.

**Figure 5:**
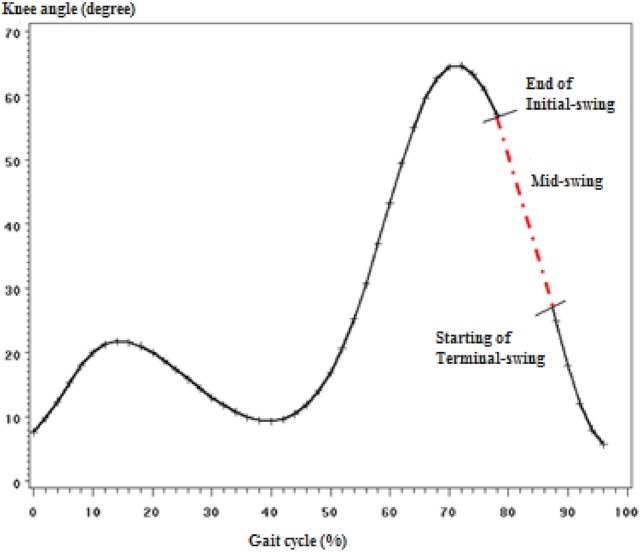
**Illustration of how the mid-swing phase connects the end of initial-swing with the start of terminal swing (knee angle curve)**.

The highlight of the finding was the results of MSw gait phase modified behavior. Manipulation of both parameters in the coupled pair that was found to have the largest mean normalized error in MSw phase did not improve the performance at that particular phase. Worse is, the manipulation of either parameter at the MSw phase caused the neighboring sub-phases (ISw and TSw) yield larger mean normalized error. This indicated biomechanical justification of the momentum and gravity influence during MSw. The prosthetic knee should be left independent to extend by the effect of momentum produced by its weight and the gravity at the mid-swing in order to connect the end of initial-swing phase with the beginning of terminal-swing as illustrated in Figure 5. This concept was discussed as “ballistic synergy” concept introduced by Pitkin that allowed for the control simplification and reduces the power supplies demand when compared with robots for which all its degrees of freedom in motion are controlled during the entire gait (Pitkin, 2010). This would make the kinematics and kinetics match with normal human ballistic gait synergy.

In this study, the results shown that altering one of the dominant parameter that was identified using manipulate cyclograms method yielded to a better performance in most of the gait sub-phases. However, it might be oversimplified considering the complexity in the dynamics of human gait. Therefore, the findings will need to be further verified by performing experiment using a micro-controller knee on a patient and the effect in the gait of the patient is analyzed.

It has been recommended in a previous study that more prospective intervention studies that take into account the multifactorial nature on the amputees walking ability should be conducted (Van Velzen et al., 2006). Thus, we proposed the use of inter-relationship modification of two-variables interaction that co-exist during a gait cycle, especially variables across two-joints which provided an insight into the effect of controlling one variable onto another variable at other joints during walking for an amputee.

Finally, only the affected limb of each subject was thoroughly investigated and reported and the actual speed of each subject was not considered. Several adaptation strategies in terms of joint power or work in both the amputated and intact leg were demonstrated in patients with transfemoral amputation (Prinsen et al., 2011). Investigation on the bilateral control was only done to determine the difference in leading or trailing limb kinematic behavior but not in more detailed toward its cyclograms changes. Further investigation would offer a more precise understanding of each subject's compensatory movement.

## Author contributions

FJ, NAH, and NMS developed the scientific content and context of the article. NMS performed the data collection of the study participants and analysis of the data. TC referred and pre-screened the study participants and contributed clinical insights into the article. NAO provided the funding, setup and facilities to perform the study and oversees the scientific content of the study. The final article was majorly written by FJ, NAH, and NMS.

## Funding

This study was funded by Ministry of Higher Education (MOHE) of Malaysia; grant no: UM.C/HIR/MOHE/ENG/10 and the first author's research work is supported by Ministry of Higher Education, Malaysia and International Islamic University Malaysia under SLAB/SLAI scholarship.

### Conflict of interest statement

The authors declare that the research was conducted in the absence of any commercial or financial relationships that could be construed as a potential conflict of interest.
